# Ecto-protein kinases and phosphatases: an emerging field for translational medicine

**DOI:** 10.1186/1479-5876-12-165

**Published:** 2014-06-12

**Authors:** Garif Yalak, Yigal H Ehrlich, Bjorn R Olsen

**Affiliations:** 1 Department of Developmental Biology, Harvard School of Dental Medicine, 188 Longwood Avenue, Boston, MA 02115, USA; 2 Program in Neuroscience, College of Staten Island (CSI), City University of New York, 2800 Victory Boulevard, Staten Island, NY 10314, USA

**Keywords:** Ecto-protein kinase, Ecto-protein phosphatase, Phosphorylation, Disease marker, Extracellular-drug targets

## Abstract

Progress in translational research has led to effective new treatments of a large number of diseases. Despite this progress, diseases including cancer and cardiovascular disorders still are at the top in death statistics and disorders such as osteoporosis and osteoarthritis represent an increasing disease burden in the aging population. Novel strategies in research are needed more than ever to overcome such diseases. The growing field of extracellular protein phosphorylation provides excellent opportunities to make major discoveries of disease mechanisms that can lead to novel therapies. Reversible phosphorylation/dephosphorylation of sites in the extracellular domains of matrix, cell-surface and trans-membrane proteins is emerging as a critical regulatory mechanism in health and disease. Moreover, a new concept is emerging from studies of extracellular protein phosphorylation: in cells where ATP is stored within secretory vesicles and released by exocytosis upon cell-stimulation, phosphorylation of extracellular proteins can operate as a messenger operating uniquely in signaling pathways responsible for long-term cellular adaptation. Here, we highlight new concepts that arise from this research, and discuss translation of the findings into clinical applications such as development of diagnostic disease markers and next-generation drugs.

## Introduction

Protein phosphorylation, a reversible posttranslational modification carried out by the enzymes protein kinases and phosphatases, is arguably one of the most critical regulatory biochemical processes operating in physiological systems. The covalent attachment of a phosphate group by a protein kinase and detachment by a protein phosphatase is rapid and energy efficient, making protein phosphorylation one of the most common post-translational modifications in cells [1]. It is thus not surprising that intracellular protein kinases have emerged as one of the most important drug targets, with some 20 drugs on the market and hundreds in clinical trials [2].

**Figure 1:**
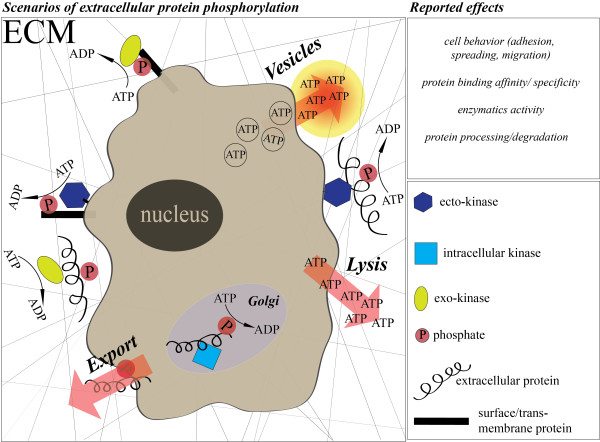
**Potential scenarios of extracellular protein phosphorylation.** Potential scenarios of extracellular protein phosphorylation are shown. Extracellular ATP can enter the extracellular matrix (ECM) through vesicle secretion or cell lysis [4]. Extracellular proteins or the extracellular domains of cell surface and trans-membrane proteins may be phosphorylated during biosynthesis and then exported, or phosphorylated after release or appearance at the cell surface by exo- and ecto-kinases, respectively.

It has been estimated that 30% of all human proteins are phosphorylated at one or another stage during their lifecycle [3]; however this percentage may be an underestimation since it does not include extracellular proteins. All preconditions for protein kinases to be active also exist in the extracellular environment, such as sufficiently high concentrations of extracellular ATP and a growing body of evidence is documenting the existence and activity of these enzymes in extracellular spaces (Figure 1; [4-7]). In fact, evidence that many prominent kinases and phosphatases are located extracellularly has been obtained *in-vivo* as well as *in-vitro* including FAM20C [7], PKA [8,9], PKC [10,11], CKII [12], alkaline phosphatase [13], tartrate-resistant acid phosphatase (TRAP) [14] and the PTEN phosphatase [15] (Table 1). A recent review provides an in-depth mining of the extensive literature in the field, as well as of high-throughput mass spectrometry data, describing known phosphorylated proteins in the extracellular matrix in many different types of healthy and diseased tissues and animal species [4]. Major components of the extracellular matrix and cell surface proteins, including fibronectin [16], vitronectin [17], osteopontin [18], collagens [19], fibrinogen [20], laminin [21], CD36 [22], β-amyloid precursor protein (APP) [23], T-cell-receptor complex (TCR) [24] and many more, have been reported to be phosphorylated in vitro and in vivo [4]. Evidence that this enzymatic regulatory activity may be important in extracellular matrix biology and pathology, cancer growth, tissue engineering, regenerative medicine, immune response, cardiovascular and neuronal biology is growing [4]. These recent findings suggest that phosphorylation of extracellular matrix proteins provides an important basis for identifying novel biomarkers and for design of new therapeutic drugs. In fact, a clinical application based on extracellular protein-phosphorylation has already been patented [25], and recent studies propose the involvement of extracellular protein kinases and phosphatases in diseases such as cancer, Alzheimer’s, cardiovascular disorders and others. In this review, we highlight the potential of this research area as an emerging new field in translational medicine and for developing novel medical applications.

**Table 1:** Reported prominent extracellular protein kinases and phosphatases

**Kinase**	**Sample**	**Reference**
PKA	serum from cancer patient	[8,9]
PKC	human platelets, hippocampal neurons	[10,26]
CKII	human prostatic cancer cell line	[12]
FAM20C	HEK293T	[7]
Alkaline phosphatase	human serum	[13]
PTEN	primary human breast tumor	[15]
TRAP	human serum	[14]

## Small differences can have huge impact

Biomolecules such as insulin or interferon serve as important drugs today. Seven of the top 10 biopharmaceuticals in 2012 were indeed biomolecular drugs, according to FiercePharma statistics. These include extracellular proteins. For example, recombinant BMP-2, an FDA approved drug, is routinely used to induce bone formation in bone defects [27]. Recombinant BMP-7 is also FDA approved as an alternative to autografts [28]. The design and production of such drugs need to be precise. Even small differences in structure can have a huge impact on their performance. The phosphorylation state of these proteins is one of these critical factors. These drugs are produced as recombinant proteins; therefore their phosphorylation state may vary from batch to batch. Our analyses show that both BMP-2 and BMP-7 can occur in phosphorylated states in human biosamples; however when they are used in the clinic, this modification is largely ignored. Paying attention to their state of phosphorylation may have significant consequences for their effective activities. Similar effects should be considered when growing cells in culture on surfaces coated with extracellular proteins; a standard method used today in basic research as well as in translational science. The phosphorylation state of matrix proteins can vary from batch to batch of purified or commercially available protein substrates for cell culture. Such variations seem minor, but they can cause significant differences in cell behavior. For example, while the phosphorylated form of the extracellular protein vitronectin promotes cell adhesion, the non-phosphorylated form inhibits cell adhesion [29]. Similarly, the phosphorylation state of extracellular proteins was reported to regulate the binding affinity as well as specificity in protein activity. For example, phosphorylation of CD36 by PKC at the position T92 leads to stronger binding to collagen, but results in loss of thrombospondin binding. The effect is reversible and suggests a regulated mechanism [22]. Finally, MMP2 enzyme activity was shown to be regulated by extracellular PKC phosphorylation, in that dephosphorylation leads to increased activity, and phosphorylation inhibits the activity [30,31]. In all translational studies and clinical developments where extracellular proteins are involved, their phosphorylation state might have substantial effects on the outcomes and needs to be taken into account.

## Impact on diseases

The activity of extracellular protein kinases has been described in the context of several physiological processes. These include leukocyte and macrophage adhesion and migration [32,33], fertilization [34], platelet function [35,36], blood coagulation [37], complement system function [38], receptor specificity and sensitivity [29], neuronal development and adaptation [39], synaptic plasticity and memory formation [40], (for a review see [4]). The involvement of extracellular protein kinases has also been reported for a number of diseases (Table 2). For example, in Alzheimer’s phosphorylation of amyloid-β-peptides by ecto-PKA at the cell surface and in the cerebrospinal fluid promotes the formation of toxic aggregates leading to increased aggregation and decreased clearance [41]. Phosphorylated amyloid-β-peptides have been detected in the brains of Alzheimer’s disease patients and this may offer new ways to target the disease. In prostate or breast cancer patients, enhanced kinase levels and activities are found in sera. It has been shown that tumor cells secrete cAMP-dependent PKA; however, the function of the secreted form is unknown [8,9,42]. Analysis of serum from a large number of Schizophrenia patients revealed 72 phosphoproteins and phosphorylation-specific changes in 59 of these proteins compared with samples from matched healthy controls [43].

**Table 2:** Extracellular phosphorylation in different diseases

**Disease**	**Kinase**	**Protein**	**Effect**	**Reference**
Alzheimer’s disease	ecto-PKA	amyloid-β-peptides	enhanced aggregation of amyloid-β-peptides	[41]
Prostate cancer	cAMP-dependent PKA			[44]
Breast cancer	cAMP-dependent PKC			[11]
Schizophrenia		72 proteins		[45]
Thrombosis and atherosclerosis		F11R aka JAM-A		[46]
Raine syndrome	FAM20C	SIBLINGs		[7]
Amelogenesis imperfecta	FAM20C	enamelin	calcium binding affected	[47]
Bacterial infection	PKC			[48]
Parasite infection	PKC, CK2			[49,50]
HIV	ectophosphatase			[51]
Memory formation	ecto-PKC			[26]

Extracellular protein phosphorylation is believed to play a role in cardiovascular disease [46,52]. For example, protein kinase C and protein kinase A have been detected on the surface of platelets. Furthermore, platelets have been shown to selectively secrete PKC isozymes as well as PKA. While their mechanisms of action have not yet been fully determined, current data suggest an important role in fibrinolysis [35,37,46,52]. A particularly well-studied example of the important function of an extracellular kinase is the Raine syndrome, caused by loss-of-function mutations in the extracellular kinase FAM20C. This leads to biomineralization defects [7]. Another well-studied example is a mutation in enamelin resulting in loss of a phosphorylation site for FAM20C. The phosphorylation at this site is necessary for binding of calcium and the loss of this calcium binding site in enamelin causes Amelogenesis Imperfecta [47]. Since the role of extracellular protein phosphorylation was barely considered in disease etiology in the past, extracellular protein phosphorylation may play a role in many more diseases than currently known.

## Novel disease markers and drug targets

The activities of extracellular protein phosphatases are routinely measured by an *alkaline phosphatase blood test* in the clinic, as a disease marker for liver diseases, bone disorders or cancer. In addition, the use of tartrate-resistant acid phosphatase (TRAP), produced by osteoclasts, is being discussed as a marker [13,14]. Similarly, an assay for extracellular protein kinases have been proposed (and recently patented) as biomarker for specific cancer types [25]. Remarkably, the activity of extracellular protein kinases in sera of melanoma patients correlates with the appearance and size of the tumor and is significantly decreased after removal of the tumor [9]. Not only cancer but also other diseases might be detected earlier by utilizing the phosphorylation state of extracellular proteins as markers. A clear difference in the phosphorylation pattern of serum proteins in sera of schizophrenia patients was mentioned above [45]. Further studies detected 502 serum proteins to be phosphorylated in sera of 80 healthy individuals and discussed the phosphorylation pattern as potential disease marker [53-55]. A large number of studies describing the activity of extracellular kinases in parasites and bacteria open the door to the discovery of novel methods for detecting and treating infections [48,49]. Further progress in this area could lead to novel and/or more reliable biomarker assays in clinical studies of cancer and other diseases and new therapies [25].

The location of extracellular protein kinases provides a unique advantage for drug development. Potential inhibitors or activators of extracellular kinases and phosphatases can be designed purposefully as lipid insoluble molecules that are unable to penetrate into cells through the plasma membrane. This may provide greater selectivity and fewer side effects, as it will prevent interaction of these drugs with intracellular kinases [56]. An excellent example of this kind of new drug would be a water-soluble, membrane-impermeable peptide or peptido-mimetic based on the sequence and tertiary structure of the region surrounding a critical phosphorylated amino-acid residue located in the extracellular domain of a specific protein substrate of an ecto- or exo-protein kinase [56].

Analyses of drug-target interactions are crucial during the drug design phase. Protein phosphorylation is known to regulate protein-protein interaction in many cases inside the cell [57]. During the design of potential drugs for targeting extracellular or cell surface proteins, the phosphorylation state of the proteins also needs to be taken into account. An example of how important the phosphorylation state of a drug may be is demonstrated by the Multiple Sclerosis drug Fingolimod (FTY720); its phosphorylation state is believed to be regulated by the ecto-phosphatase LPP3 in vivo [58].

## A novel concept revealed by extracellular protein phosphorylation

In several cell types ATP is stored within secretory vesicles and released by exocytosis upon cell-stimulation. Repetitive, high frequency stimulation of these cells produces unusually high concentrations of ATP in the extracellular space, and particularly in synaptic-clefts between neurons. Such events do not occur usually during the course of routine cellular communications. Instead, they represent unique biochemical signals responsible for triggering adaptive processes with long-lasting consequences [59]. In the next step, extracellular protein kinase activity that is dependent on these higher ATP concentrations produces a phosphorylation-dependent event required for the induction of long-term adaptive changes [39,56,59,60]. Platelet and neuronal activities have been most studied in regard to this aspect of extracellular phosphorylation. Massive stimulation of localized platelets produces high concentrations of extracellular ATP during blood coagulation and atherosclerotic plaque formation, and extracellular protein kinase activities have been implicated in both [56]. High frequency stimulation of neurons in the hippocampus induces a memory-related adaptive process called long term potentiation (LTP), involving high ATP concentrations in the synaptic-cleft [60]. LTP is a physiological measure of memory formation in the brain that requires an extracellular protein kinase C activity [26]. Similarly, the same concept suggests that high levels of extracellular ATP produced by neurons during seizures may trigger extracellular phosphorylation activity involved in the etiology of Epileply.

## Conclusions and future outlook

Recent advances in the research of reversible extracellular protein phosphorylation activity provide unique opportunities for translational medicine to design novel drugs and identify new disease markers. While the mechanisms of action of extracellular protein kinases and phosphatases are being uncovered and complete knowledge of their physiological roles requires further investigation, clinical studies already show their potential as novel biomarkers and drug targets in diseases such as cancer [44], neuronal [5,39,41,56] and cardiovascular disorders [10,33,46,52]. Initial studies and patents show encouraging results regarding the performance of such biomarkers compared to conventional markers [25]. The fact that secreted alkaline phosphatase is routinely used as a biomarker in clinics today suggests that other secreted protein kinases and phosphatases will be used as novel markers in the future [61]. A large number of extracellular proteins have been reported in phosphorylated states as a result of interactions with yet unknown extracellular protein kinases. It is thus expected that many more active kinases will be discovered in the extracellular environment. Finally, more directed studies may uncover the involvement of extracellular phosphorylation in many more diseases than those that are currently known and open up new avenues for novel therapies.

## Abbreviations

TRAP: Tartrate-resistant acid phosphatase; PKA: Protein kinase A; PKC: Protein kinase C; ATP: Adenosine triphosphate; APP: β-amyloid precursor protein; LTP: Long term potentiation; TCR: T-cell-receptor complex; BMP: Bone morphogenetic protein; FAM20C: Family with sequence similarity 20; CD36: Cluster of differentiation 36.

## Competing interests

The authors declare that they have no competing interests.

## Authors’ contributions

All authors have contributed equally. All authors performed article searches, drafted and revised the manuscript. All authors read and approved the final manuscript. Author BR Olsen provided financial support for publication.
